# *OsMFS1*/*OsHOP2* Complex Participates in Rice Male and Female Development

**DOI:** 10.3389/fpls.2020.00518

**Published:** 2020-05-15

**Authors:** Jiayu Lu, Chaolong Wang, Haiyu Wang, Hai Zheng, Wenting Bai, Dekun Lei, Yunlu Tian, Yanjia Xiao, Shimin You, Qiming Wang, Xiaowen Yu, Shijia Liu, Xi Liu, Liangming Chen, Ling Jang, Chunming Wang, Zhigang Zhao, Jianmin Wan

**Affiliations:** ^1^State Key Laboratory for Crop Genetics and Germplasm Enhancement, Jiangsu Plant Gene Engineering Research Center, Nanjing Agricultural University, Nanjing, China; ^2^National Key Facility for Crop Gene Resources and Genetic Improvement, Institute of Crop Science, Chinese Academy of Agricultural Sciences, Beijing, China

**Keywords:** meiosis, sterile, *OsMFS1*, *OsHOP2*, rice

## Abstract

Meiosis plays an essential role in the production of gametes and genetic diversity of posterities. The normal double-strand break (DSB) repair is vital to homologous recombination (HR) and occurrence of DNA fragment exchange, but the underlying molecular mechanism remain elusive. Here, we characterized a completely sterile *Osmfs1* (male and female sterility 1) mutant which has its pollen and embryo sacs both aborted at the reproductive stage due to severe chromosome defection. Map-based cloning revealed that the OsMFS1 encodes a meiotic coiled-coil protein, and it is responsible for DSB repairing that acts as an important cofactor to stimulate the single strand invasion. Expression pattern analyses showed the *OsMFS1* was preferentially expressed in meiosis stage. Subcellular localization analysis of OsMFS1 revealed its association with the nucleus exclusively. In addition, a yeast two-hybrid (Y2H) and pull-down assay showed that OsMFS1 could physically interact with *OsHOP2* protein to form a stable complex to ensure faithful homologous recombination. Taken together, our results indicated that *OsMFS1* is indispensable to the normal development of anther and embryo sacs in rice.

## Introduction

Meiosis is indispensable for sexual reproduction in eukaryotes, and is a specialized cellular division process. In this process, the sexually reproducing cells undergo a round of DNA replication and two successive rounds of nuclear division, and ultimately form genetically recombined haploid generative cells (Dawe, 1998; Kleckner, 2006). During the first division (meiosis I), homologous pairing and synapsis form a synaptonemal complex (SC, a tripartite proteinaceous structure), then carry on genetic recombination and chromosomal segregation. During the second division (meiosis II), the sister chromatids separate equally to form four genetically different haploid cells. Compared with mitosis, these two chromosome segregations of meiosis possess a great amount of innovations (Ma, 2006; Mercier et al., 2015; Gray and Cohen, 2016).

Although there are many reports on meiosis (Keeney et al., 1997; Fernandez-Capetillo et al., 2003; Zhang et al., 2017), the molecular mechanism of homologous recombination (HR) remains uncovered (Wang and Copenhaver, 2018). HR possesses an important biological significance within the coexistence of high-risk chromosomal behavior which can lead to chromosomal developmental defects and endanger the survival of the organisms. Therefore, it is crucial to ensure the smooth progress of HR. HR initiates with double-strand breaks (DSBs), which are induced by Spo11 (a conserved topoisomerase-like protein) during the early stages of meiosis (Bergerat et al., 1997; Keeney et al., 1997; Shehre-Banoo et al., 2007). Subsequently, the DSBs are processed to form recombinogenic single-stranded tails with 3′ termini (Farah et al., 2005; Mimitou and Symington, 2009), which serves as a probe for researching a homologous repair template. The extensive single-stranded DNAs (ssDNA) were bound by RPA (heterotrimeric replication protein) complexes with high affinity to protect them from nuclease (Paques and Haber, 1999; Lee et al., 2003; Aklilu et al., 2014). *RAD51* and *DMC1* are two homologs of the bacterial recombinase RecA (Sung and Robberson, 1995; Shinohara et al., 1997; Hong et al., 2001). DMC1 and RAD51 proteins were recruited to replace the RPA complex (Ellen et al., 2006) and form a right-handed helical nucleoprotein filament on the single strands in an ATP-dependent manner to promote the search for homology and catalyze the single strand invading homologous duplex DNA (Tashiro et al., 1996; Hong et al., 2001; Masson and West, 2001; Cloud et al., 2012; Marie-Therese et al., 2012). The strand invasion leads to some or all DSBs forming homologous joint molecules, which were termed the SC (Schwacha and Kleckner, 1997; Masson et al., 1999; Masson and West, 2001; Sheridan et al., 2008), and finally produce crossovers (COs) or non-crossover (non-COs) due to different resolutions (Allers and Lichten, 2001; Hunter and Kleckner, 2001; Borner et al., 2004; Mimitou and Symington, 2009).

The molecular, genetic, and molecular phylogenetic analysis indicated that many meiotic proteins are conserved among plants, animals, and fungi (Gray and Cohen, 2016; Loidl, 2016). The HR pathway requires auxiliary proteins to assist the strand invasion of the recombinase DMC1 and RAD51, with the proteins MND1 (meiotic nuclear division protein 1) and HOP2 (homologous-pairing protein 2) acting as vital cofactors (Leu et al., 1998; Patrick et al., 2003; Yi-Kai et al., 2004; Zierhut et al., 2004; Neale and Scott, 2006). The MND1 protein was first identified in screening for genes in budding yeast, and the disruption of MND1 would lead to meiotic arrest, aberrant synapses, and defects in SC formation (Rabitsch et al., 2001; Zierhut et al., 2004). Previous studies reported that MND1 could physically interact with HOP2 to form a heterodimeric complex (Hideo and Shirleen, 2002; Li et al., 2004; Yi-Kai et al., 2004; Julien et al., 2007; Zhao et al., 2014). MND1 and HOP2 could be coimmunoprecipitated from crude extracts of meiotic yeast cells (Hideo and Shirleen, 2002). The *Arabidopsis mnd1* and *hop2* mutant plants were disrupted in both male and female meiosis due to them failing to form bivalents (Claudia et al., 2006; Stronghill et al., 2010). Biochemical studies have shown that the MND1/HOP2 complex can condensate dsDNA (double-stranded DNA) and physically interact with DMC1 and RAD51 that efficiently stabilize the presynaptic filament and stimulate strand invasion activity (Petukhova et al., 2003; Yi-Kai et al., 2004; Johnson and O’Donnell, 2005; Vignard et al., 2007; Pezza et al., 2010). However, neither MND1 nor HOP2 can facilitate this reaction alone (Pezza et al., 2010). In budding yeast, the *hop2* and *mnd1* mutants exhibited the same defects in meiosis and suggested that MND1/HOP2 complex was the central role during the HR process (Leu et al., 1998; Gerton and Derisi, 2002; Petukhova et al., 2003; Zierhut et al., 2004). However, related research in monocots has not been reported.

Here we identified a completely sterile *Osmfs1* (male and female sterility 1) mutant at the heading stage. Our results suggested that the heterodimeric complex OsMFS1/*OsHOP2* is vital for the repairment of DSBs in rice.

## Materials and Methods

### Plant Materials and Growth Conditions

We identified one completely sterile mutant by screening ethyl methanesulfonate (EMS)-mutagenized M_2_ rice lines. The F_2_ mapping population was generated from a cross between the heterozygous mutant and N22 (*indica*). The F_2_ population was grown in the Tu Qiao Experiment Station of Nanjing Agricultural University. At the mature stage, plants with the phenotype of complete sterility were selected as homozygous mutants for mapping.

### Preparation of Embryo Sacs

We obtained embryo sacs at different stages by fixing WT and mutant florets in Carnoy’s solution (75% ethanol and 25% acetic acid), and the dissected ovaries were preexisted in 70% ethanol. Subsequently, the samples were hydrated sequentially in 50, 30, and 15% ethanol and distilled water, stained with 1% eosin-Y for about 8 h, and washed several times in distilled water until colorless. The samples were treated for 8 h in citric acid-disodium hydrogen phosphate buffer (0.1 mol/l, pH 5.0) and followed by Hoechest staining (25°C in darkness for 24 h). The samples were washed three times in distilled water, and processed through an ethanol series (15, 30, 50, 70, 85, 95, and 100%) for dehydration. Then, the samples were treated in 1:1 ethanol and methylsalicylate for 1 h, cleared three times in methylsalicylate (2, 2, 15 h), and finally saved in methylsalicylate. We applied a laser confocal scanning microscope (Zeiss Microsystems LSM 700) to compare the various developmental stages of embryo sacs between the WT and the *Osmfs1*.

### Meiotic Chromosome Examination

Young panicles (40∼60 mm) of WT and the mutants were fixed in Carnoy’s solution (ethanol: glacial acetic 3:1) and stored at −20°C until needed. Firstly, we selected one of six anthers to stain with 1% acetocarmine solution on glass slides, and then judged the development stage with optical microscopy. Finally, the appropriate anthers at the meiotic stage were squashed under a cover slip in 40% acetic acid. The slides were frozen in liquid nitrogen for 5 min and dried at room temperature after quickly removing the cover slips. After that, the samples were treated with 20 μl of 0.1 mg ml^–1^ propidium iodide to stain chromatin for 20 min. The male meiotic chromosomes were observed using a fluorescence microscope.

### Scanning (SEM) and Transmission Electron Microscopy (TEM)

The anthers of WT and mutants were fixed in 2.5% glutaraldehyde, rinsed three times using distilled water dehydrated through an ethanol series (30, 50, 70, 85, 90, 100, and 100%), fixed in 1% OsO_4_ for 2-hour, dehydrated through an ethanol series, and subjected to critical point drying with CO_2_. The anthers were coated with gold by an E-100 ion sputter and observed with a scanning electron microscope (S3400; Hitachi). For TEM, mature anthers were fixed in 1% glutaraldehyde and 1% OsO_4_ for 1-hour and dehydrated through an ethanol series. The samples were embedded in Spurr’s medium prior to thin sectioning. Sections were double-stained with 2% uranyl acetate and 2.6% aqueous lead citrate solution, and examined with a JEM-1230 transmission electron microscope (Jeol) at 80 kV (Ren et al., 2014; Wang et al., 2015).

### Map-Based Cloning

We identified the *Osmfs1* mutant, which was mutagenized from Ninggeng 4 (a *japonica* variety) by ethyl methanesulfonate (EMS). The F_2_ population was generated from a cross between *Osmfs1* heterozygous plants and N22 (an *indica* variety), and finally 111 completely sterile plants were selected from 1500 F_2_ population. The DNA of these plants were isolated from leaves using the modified CTAB method (Murray and Thompson, 1980), and the gene was fine-mapped to a 282-kb genomic region between the markers NN9S-L4 and NN9S-L7. Sequence analysis showed that the *Osmfs1* mutants existed a premature translational termination. For fine mapping of the *Osmfs1* locus, bulked-segregated analysis was used and molecular markers were designed by comparison of the local genomic sequence differences between N22 (*O. sativa*, *indica*) and Nipponbare (*O. sativa*, *japonica*), available at the National Center for Biotechnology Information (NCBI)^1^. Primers used in the mapping are listed in Supplementary Table S1.

### CRISPR-Cas9 Vector Construction

For the CRISPR-Cas9 vector, two 20-bp target sites specific for *MFS1* and *HOP2* were synthesized^2^, fused with the *Aar*I linearized intermediate vector SK-Grna, and then introduced into CRISPR-Cas9 binary vector pCAMBIA1305 (Sun et al., 2017) to generate a knock-out construct. The sequencing confirmed plasmids were transformed into A. tumefaciens strain EHA105, and we used *Agrobacterium tumefaciens*-mediated transformation of rice callus (T65, *O. sativa*, *japonica*) to generate transgenic rice plants (Jeon et al., 2000). We mixed two kinds of *Agrobacterium* (*MFS1* and *HOP2*) in equal amounts to generate double mutant plants. Primers used in the plasmid construction are listed in Supplementary Table S1.

### β-Glucuronidase (GUS) Histochemical Staining

We amplified a putative 2.6-kb genomic promoter fragment by PCR and cloned the fragment into the binary vector pCAMBIA1305 digested with *Bam*HI and *Hin*dIII by In-Fusion (Takara Bio, Japan) to drive *GUS* reporter gene expression (primers used in the plasmid construction are listed in Supplementary Table S1). The construct was transformed into T65 (*O. sativa, japonica*) by *Agrobacterium tumefaciens*-mediated method. Young spikelets of T_1_ transgenic plants were incubated in GUS staining solution as described previously (Jefferson, 1987). The samples were observed under the stereomicroscope.

### Subcellular Localization of OsMFS1 Protein

To investigate the cellular localization of the OsMFS1 protein, the *OsMFS1* cDNA was fused with GFP and inserted in the pAN580-GFP vector or pCAMBIA1305.1-GFP between the cauliflower mosaic virus (CaMV) 35S promoter and the nopaline synthase (NOS) terminator (primers sequences are listed in Supplementary Table S1). The *35S-OsMFS1-GFP* plasmid was transformed into rice protoplasts and *N. benthamiana* epidermal cells according to the protocols described (Zhao et al., 2013).

### RNA Isolation and Quantitative real-time reverse transcription-PCR (RT-qPCR)

Total RNA was extracted using a RNeasy Plant Mini Kit (Qiagen) from various tissues and anthers at different stages. The first-strand cDNA was synthesized using 1 μg RNA and QuantiTect^®^ Reverse Transcription Kit (Qiagen). For RT-qPCR analyses, 20 μl reaction volumes containing 0.4 μl of cDNA, 0.2 μM of gene-specific primers, and SYBR Premix Ex Taq Kit (TaKaRa), running on ABI Prism 7900 HT Sequence Detection System (Applied Biosystems) according to the manufacturer’s instructions and three biological repeats were performed. The rice *Ubiquitin* gene was used as the internal control. Primers used for RT-qPCR are listed in Supplementary Table S1.

### Yeast Two-Hybrid (Y2H) Assay

Full-length cDNA of genes were amplified and cloned into the Y2H prey vector pGADT7 or bait vector pGBDT7 (Clontech). Then the two plasmid types were co-transformed into Gold yeast (*Saccharomyces cerevisiae*), and transformant plants were grown on SD–Leu/–Trp plates at 30°C for 3 days. Subsequently, six individual clones were selected and mixed into 60 μl of 0.9% NaCl and diluted 10, 100, and 1000-fold, each concentration absorbing 6 μl. The activation ability was assayed on selective media -LTHA (SD–Leu/–Trp/–His/–Ade) containing X-α-gal (40 μg ml^–1^).

### Pull-Down (*in vitro*) Assay

The coding regions of *OsMFS1* and *OsHOP2* were amplified and *OsMFS1* was cloned into the pMAL-c2X vector while *OsHOP2* was cloned into the pCOLD-His vector, which carried an N-terminal His Tag. Then we obtained MBP-OsMFS1 and pCOLD-His-*OsHOP2* plasmids. Then the two plasmids transformed into *Escherichia coli* BL21 cells. MBP- and MBP-OsMFS1-coupled beads were used to capture pCOLD-His-*OsHOP2*. The western blot was applied to analyze the results of pull-down.

### Accession Numbers

Sequence data from this article for the mRNA, cDNA, and genomic DNA can be found in the GenBank/EMBL/Gramene data libraries or Web site under accession numbers: *OsMFS1*, *Os09g0280600*; *OsHOP2*, *Os03g0710100*; *OsDMC1A*, *Os12g0143800*, *OsDMC1B*, *Os11g0146800*, *OsRAD51-A1*, *Os11g0615800*; *OsRAD51-A2*, *Os12g0497300*; *OsRPA2b*, *Os02g0633400*; *ATMND1*, and *AT4G29170*.

## Results

### Morphological Characterization of the *Osmfs1* Mutant

**FIGURE 1:**
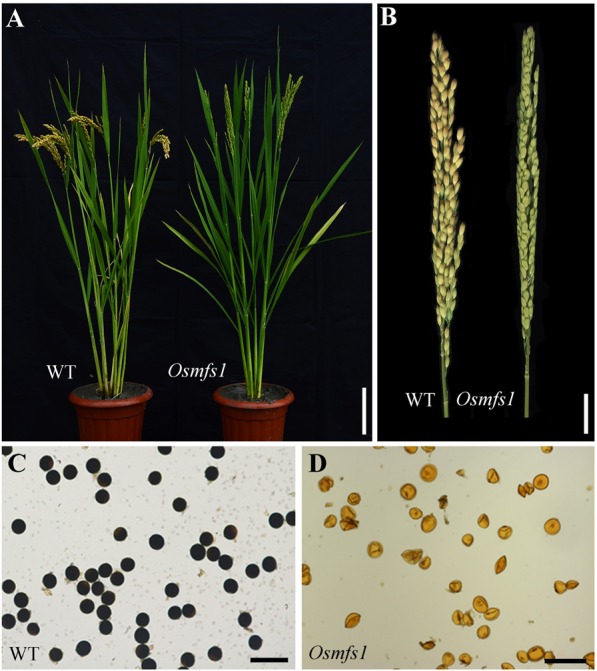
Phenotype analysis between the WT and the *Osmfs1* mutant. **(A)** Comparison between a WT plant (left) and an *Osmfs1* mutant (right) plant at the heading stage. Bars = 10 cm. **(B)** Comparison between a WT panicle (left) and an *Osmfs1* mutant panicle (right) at the harvest stage. Bars = 2 cm. **(C)** Viable pollen grains in a WT plant. **(D)** Inviable pollen grain in an *Osmfs1* mutant plant. Bars = 100 μm in panels **(C,D)**.

At the seedling, tillering, and flowering stages, the *Osmfs1* mutant showed no obvious differences from the WT. At anthesis, however, mutants exhibited complete sterility compared with WT plants (Figures 1A,B), while heterozygous plants appeared fully fertile. I_2_-KI staining indicated that the *Osmfs1* mutant pollen grains lacked starch and were completely non-viable when compared with the WT (Figures 1C,D). Then we detected the female fertility of the *Osmfs1* mutant by pollinating the WT pollens, and the mutant failed to set seeds (Supplementary Figure S1F). Therefore, we concluded that the *Osmfs1* mutant is sterile in both male and female instances.

### Abnormal Development of Male Gametes in the *Osmfs1* Mutant

**FIGURE 2:**
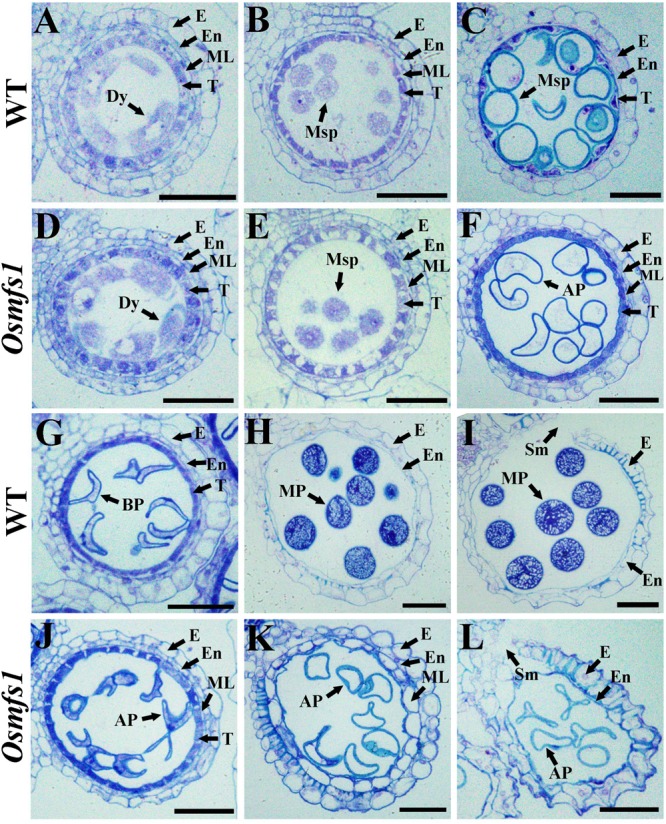
Semi-thin section comparison of anther development in the WT and the *Osmfs1* mutant. Transverse section of WT and *Osmfs1* anthers were stained with toluidine blue. The images are of cross sections through single locules and pictures are captured with a 40× ordinary optical microscope. **(A–C)** and **(G–I)** the WT anthers; **(E,F,J**,**L)** the *Osmfs1* anthers; **(A,D)** Meiosis stage. **(B,E)** Early microspore stage. **(C,F)** Vacuolated pollen stage. **(G,J)** Bicellular pollen stage. **(H,K)** Mature pollen stage. **(I,L)** Anther dehiscence stage. E, epidermis; En, endothecium; ML, middle layer; T, tapetum; Dy, dyad cell; Msp, microspore; BP, bicellular pollen; AP, abnormal pollen; MP, mature pollen; Sm, stomium. Bars = 1 μm.

To characterize the developmental defects of pollen in the *Osmfs1* mutant, we examined anther development with semi-thin sections. During the early microspore and meiosis stage, the male meiocytes seemed to undergo normal development and no obvious difference appeared between the WT and the mutant (Figures 2A,B,D,E). During the vacuolated pollen stage, almost all wild-type vacuolated microspores regularly arranged in anther locule, and the microspores were spherical and filled with cell sap (Figure 2C). Nevertheless, the microspores of the *Osmfs1* mutant appeared with an irregular shape and no vacuoles (Figure 2F). At the bicellular pollen stage, pollen grains were shown to be crescent-shaped and cell sap ran off, preparing for the next starch filling and indiscernible difference between WT and *Osmfs1* mutant (Figures 2G,J). Mature pollen grain from the WT filled with starch granules and stained densely (Figures 2H,I), whereas mature pollen grains of the *Osmfs1* mutant lacked starch granules and showed an irregular shape (Figure 2K). The development processes of epidermis, endothecium, and tapetum were indistinguishable between the WT and the *Osmfs1* mutant (Figures 2A–G,J). At the vacuolated pollen stage, the middle layer completely degraded in WT (Figure 2C), while it slowly delayed in the mutant until the stage of anther dehiscence (Figures 2F,J,K,L).

**FIGURE 3:**
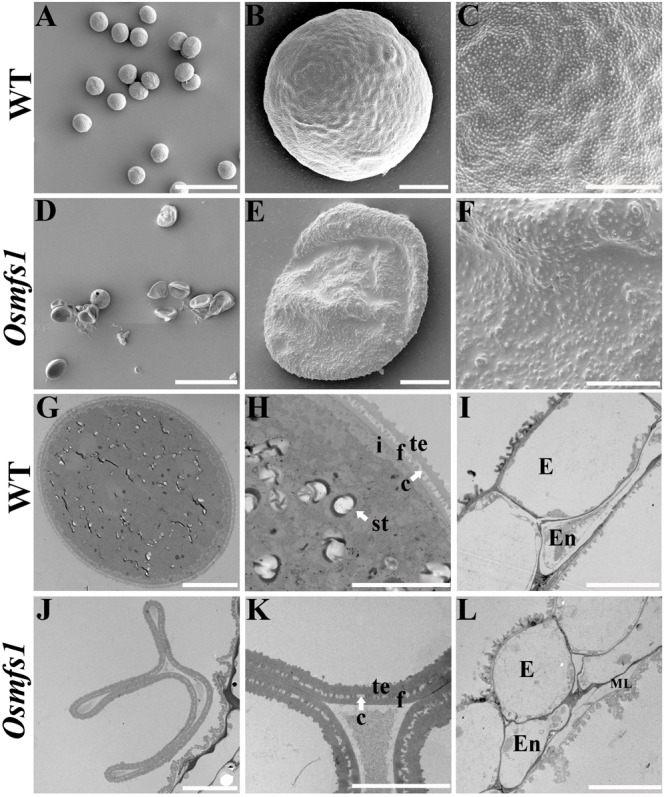
Scanning electron micrography (SEM) and transmission electron micrography (TEM) of pollen from the WT and *Osmfs1* mutant. **(A)** Scanning electron microscopy image of the mature WT pollen grain. **(B)** Magnified single pollen grain from panel **(A)**. **(C)** Further magnification showing the exine from panel **(B)**. **(D)** Scanning electron microscopy image of the mature *Osmfs1* pollen grain. **(E)** Magnified single pollen grain from panel **(D)**. **(F)** Further magnification showing the exine from panel **(E)**. **(G)** Transmission electron microscopy image of the WT pollen. **(H)** Magnification of the pollen wall from panel **(G)**. **(I)** Transmission electron microscopy image showing the WT anther wall. **(J)** Transmission electron microscopy image of the *Osmfs1* pollen. **(K)** Magnification of the pollen wall from panel **(J)**. **(L)** Transmission electron microscopy image showing the *Osmfs1* anther wall. c, columella; st, starch; i, intine; f, foot layer; te, tectum; E, epidermis; En, endothecium; ML, middle layer. Bars = 100 μm in panels **(A,D)**, 10 μm in panels **(B,E,G,J,I,L)**, and 5 μm in panels **(C,F,H,K)**.

In order to get insight into abnormalities of the *Osmfs1* pollens, SEM and TEM observation were performed. SEM observation showed that the mature pollen grains of WT were spherical and the exine of mature pollen was covered with sporopollenin (Figures 3A–C). However, pollen grains of the mutants were irregular, shrunken, and coated with less sporopollenin compared with that of WT (Figures 3D–F). The pollen wall was made up of exine and intine, and then the exine was divided into the tectum and foot layer linked by the columella. TEM indicated that the mature pollen grains of WT were filled with starch granules (Figures 3G,H). In contrast, there is no starch granule accumulation in pollen grains of the *Osmfs1* mutant instead of several degrading substances. Meanwhile, the pollen wall of *Osmfs1* mutant was dysplastic, with a thicker tectum and foot layer, and columella was degrading (Figures 3J,K). In addition, consistent with semi-thin sections, a residual middle layer still exists in the *Osmfs1* mutant while not in WT (Figures 3I,L). In conclusion, the *Osmfs1* mutant was male sterile due to the abnormal pollen development.

### Arrested Development of Embryo Sac in the *Osmfs1* Mutant

**FIGURE 4:**
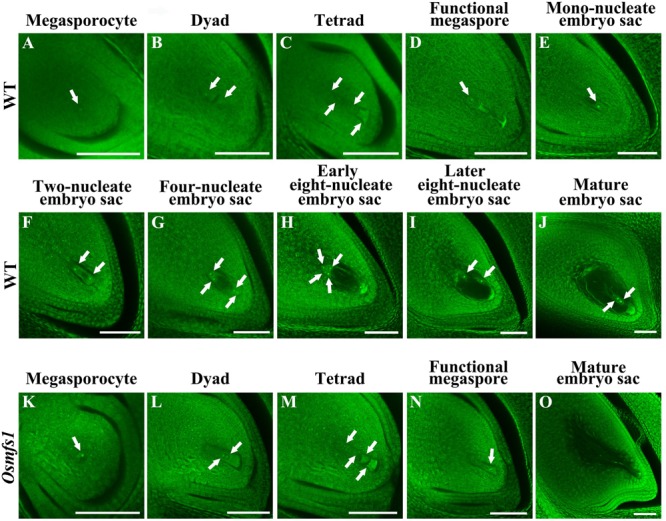
Embryo sac development in the WT and the *Osmfs1* mutant. **(A–J)** the WT, **(K–O)** the *Osmfs1*, **(A,K)** Megasporocyte, **(B,L)** Dyad, **(C,D)** Tetrad, **(D,N)** Functional megaspore, **(E)** Mono-nucleate embryo sac, **(F)** Two-nucleate embryo sac, **(G)** Four-nucleate embryo sac, **(H,I)** Eight-nucleate embryo sac, **(J,O)** Mature embryo sac. The mature embryo sacs of the WT eventually formed viable egg cells when mutant showed hollow. Scale bar = 20 μm.

When *Osmfs1* mutant pollinated with WT pollen, it failed to set seed and indicated that the embryo sac was dysplastic (Supplementary Figure S1). To further verify the cause of abortion of *Osmfs1* mutant embryo sacs, whole-mount stain-clearing confocal laser microscopy (WCLSM) was applied to investigate the development of embryo sacs of the WT and *Osmfs1* mutant. In the WT, the megasporocyte underwent meiotic divisions to produce four haploid megaspores, and three micropylar megaspores degenerated while the chalazal-most megaspore became a functional megaspore (Figures 4A–D). The size and vacuoles of the functional megaspore increased during the mono-nucleate embryo sac stage (Figure 4E), then the mono-nucleate embryo sac underwent cell division without cytokinesis to form a two-nucleate embryo sac (Figure 4F), and the two daughter nuclei migrated to the poles and a central vacuole formed (Figure 4G). Associated with the vacuole increasing in size, eight-nucleate embryo sacs were formed after two rounds of mitosis (Figure 4H). At the eight-nucleate embryo sac stage, four nuclei near the chalaza differentiated into the polar nuclei and three antipodal cells while four nuclei at the micropylar end turned into the polar nuclei, an egg cell, and two synergid cells (Figure 4H). Thereafter, the two polar nuclei expanded and migrated to the center of the embryo sac and formed a diploid central nucleus, then the central cell moved toward the egg apparatus (Figure 4I). At the mature embryo sac stage, the starch granules around the egg cell disappeared and the largest volume appeared in the embryo sac (Figure 4J).

The normal megasporocyte could be detected in the *Osmfs1* mutant (Figure 4K), and the megasporocyte went through meiotic divisions to produce dyad and tetrad (Figures 4L,M), whereas three chalazal-most megaspores degenerated at tetrad stage and the micropylar megaspore could be observed at the functional megaspore stage (Figure 4N). Thereafter, the abnormal functional megaspore was unable to undergo mitotic divisions and completely degenerated before the mono-nucleate embryo sac stage, resulting in the failure to form seven cells and eight nuclei embryo sacs (Figure 4O). Therefore, our results suggested that the development of embryo sacs of *Osmfs1* was also disturbed during the meiosis stage and resulted in the embryo sacs abortion.

### *Osmfs1* Mutant Shows Disrupted Chromosome Behavior

**FIGURE 5:**
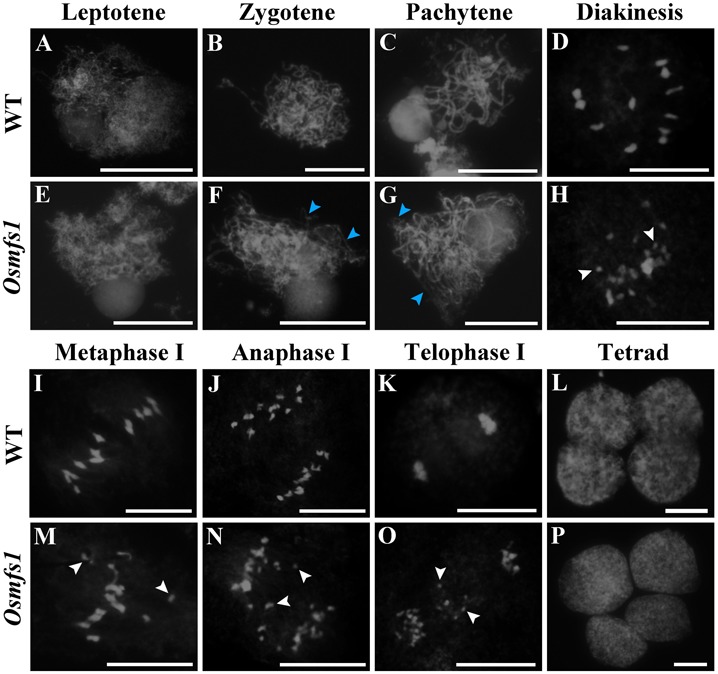
Meiotic chromosomes spreads of pollen mother cells in the WT and the *Osmfs1* mutant. **(A–D, I–L)** WT. **(E–H, M–P)** *Osmfs1*. **(A,E)** Leptotene. **(B,F)** Zygotene. **(C,G)** Pachytene stage. **(D,H)** Diakinesis. **(I,M)** Metaphase I. **(J,N)** Anaphase. **(K,O)** Telophase. **(L,P)** Tetrad. The blue arrows point to the unpaired chromosomes, the white arrows point to univalents. Bars = 100 μm.

To further investigate male sterility in the *Osmfs1* mutant, meiotic chromosome behavior was observed. In WT, condensing chromosomes were seen in single threads at leptotene when DSBs were prepared for homologous recombination (Figure 5A). At zygotene, homologous chromosomes started to pair with synapsis and were concentrated to one side of the nucleus (Figure 5B). During pachytene, all the synapsed chromosomes were scattered in the nucleus when the SC accomplished, meanwhile the strand exchange occurred between the sister chromatids of homologous chromosomes (Figure 5C). At diakinesis, chromosomes further condensed and appeared to have rod-like structures, ultimately forming twelve bivalents in the nucleus (Figure 5D). At metaphase I, the twelve bivalents aligned on the equatorial plate (Figure 5I). Subsequently, homologous chromosomes separated and migrated to opposite poles at anaphase I (Figure 5J). At telophase I, chromosomes reached opposite poles and started to condense, then cell division led to the formation of dyads (Figure 5K). Finally, four daughter cells came out after the second meiotic division (Figure 5L).

In the *Osmfs1* mutant, no obvious differences were found at leptotene compared with the WT plants (Figure 5E). However, some unpaired chromosomes could be detected during zygotene and pachytene (Figures 5F,G). The disruption was more remarkable at diakinesis, and more than 12 chromosomes caused by incomplete pairing of chromosomes dispersed among the cells, compared with 12 chromosomes in the WT (Figures 5D,H). A majority of univalents could be observed within the nucleus (Figure 5H,M), and a lot of chromosomes would not be drawn toward the poles by spindle fibers instead of scattered in cells randomly (Figures 5N,O). Finally, chromosomes were unequally separated and this led to the abnormal tetrad (Figure 5P). Therefore, we confirmed that the disorganized chromosome behavior in the meiosis stage was the key reason for the abnormal microspores.

### Isolation of *OsMFS1* Gene by Map-Based Cloning

**FIGURE 6:**
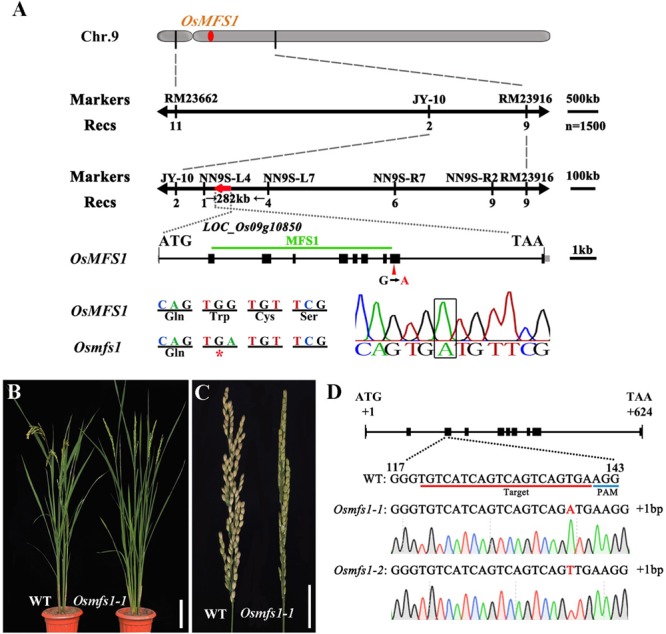
Map-based cloning of the *OsMFS1* gene and phenotype comparisons between the WT and the *Osmfs1* mutant (The positive T_3_ plants of CRISPR/Cas9). **(A)** Primary mapping was performed using markers RM23662 and RM23916 on chromosome 9, and further fine-mapped to a 282-kb genomic region between the markers NN9S-L4 and NN9S-L7, the *OsMFS1* gene was located in the region. **(B)** Comparison between a WT plant (left) and a positive plant of *Osmfs1* (right) at the heading stage. Bars = 10 cm. **(C)** Comparison between a panicle of WT (left) and a panicle of *Osmfs1* (right) at the harvest stage. Bars = 2 cm. **(D)** A schematic representation of the gene structure of *OsMFS1*, two lines with different mutation types were obtained, the sequences of both *Osmfs1-1* and *Osmfs1-2* have a single nucleotide insertion.

To determine the molecular mechanism of the mutant, we isolated the *OsMFS1* gene using a map-based cloning approach. An F_2_ population was generated from a cross between *Osmfs1* heterozygous plants and N22, and the *OsMFS1* locus was preliminarily mapped to a region between two simple sequence repeat (SSR) markers RM23662 and RM23916 on the chromosome 9. Subsequently, the location was narrowed to a 282-kb genomic region between the markers NN9S-L4 and NN9S-L7 by using 1500 F_2_ plants (Figure 6A). Sequencing and comparison analysis revealed a single nucleotide substitution of guanine (G) to adenine (A) in the 9th exon of *LOC_Os09g10850*, which is predicted to encode a meiotic coiled-coil protein. The mutation caused a frameshift that resulted in premature translational termination (Figure 6A). The *LOC_Os09g10850* gene contains ten exons and nine introns and expects a 23.9 KDa protein with 207 amino acids. We obtained sequences from the NCBI database and aligned them using Bioxm software, and the result showed that the OsMFS1 protein was conserved in several species (Supplementary Figure S2). To further verify that *LOC_Os09g10850* was the objective gene, we performed targeted mutagenesis of the *LOC_Os09g10850* in the genetic background of T65 using the CRISPR/Cas9 technology. We obtained two independent Cas9-free homozygous mutants, which were mutated at different sites. Two contained 1-bp insertion in the extron (*Osmfs1-1* and *Osmfs1-2*), and both had premature terminations of protein translation (Figures 6B–D). Like the *Osmfs1* mutant, we did not observe any significant differences between the transgenic lines and WT plants during vegetative growth, and all lines were completely sterile at the harvest stage (Supplementary Figure S1). Taken together, our results demonstrated that *LOC_Os09g10850* was the target gene of causal mutation of *Osmfs1*.

### Expression Pattern and Subcellular Localization of *OsMFS1*

**FIGURE 7:**
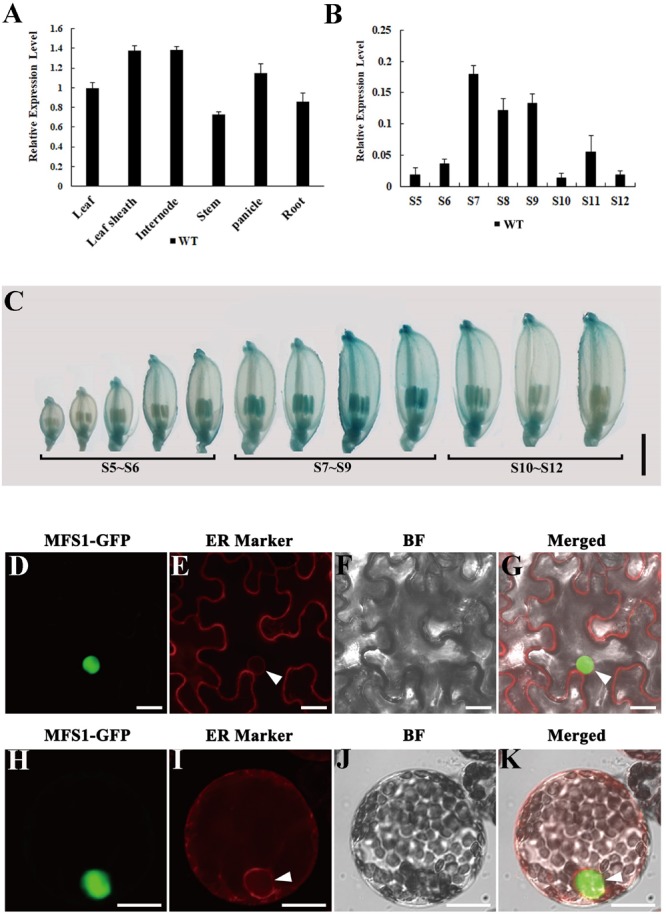
Expression pattern analysis of *OsMFS1* and subcellular localization. **(A)** The result of quantitative RT-PCR of the *OsMFS1* in various different tissues of the WT. **(B)** The result of quantitative RT-PCR of the *OsMFS1* in different stage of WT anther. The results of panels **(A,B)** were performed with three biological repeats. **(C)** Indicating the expression stages of the panicles of *OsMFS1* by GUS staining. Bars = 2 mm. **(D–K)** Subcellular localization of OsMFS1 protein in *N. benthamiana*. **(D–G)** Subcellular localization of the OsMFS1-GFP protein in the cells of leaf epidermal. **(H–K)** Subcellular localization of the OsMFS1-GFP protein in protoplasts. **(D,H)** OsMFS1-GFP is detected in the nucleus. **(E,I)** Localization of the ER marker. **(F,J)** images of Bright field. **(G,K)** Merged the images of panels **(D–F, H–J)**, respectively. Bars = 10 μm.

Quantitative RT-PCR (RT-qPCR) was performed to examine the *OsMFS1* expression in various tissues of the WT including leaves, leaf sheaths, internodes, stems, panicles, and roots. The results showed that *OsMFS1* was constitutively expressed in the above-mentioned tissues (Figure 7A). Interestingly, *OsMFS1* expressions were highly expressed in the anthers during the meiosis stage and then declined toward maturation (Figure 7B). Subsequently, we obtained transgenic plants which carried an *OsMFS1* pro:GUS vector, and the GUS signals were strongly detected in the anthers of young panicles and peaked at the meiosis stage (S7∼S9) in the transgene plants (Figure 7C), and these results were consistent with the data of RT-qPCR. In conclusion, we confirmed that the *OsMFS1* was highly expressed during the meiosis stage. In order to determine the subcellular localization of the OsMFS1 protein, we constructed a GFP (green fluorescent protein) fusion vector named OsMFS1-GFP, and the OsMFS1-GFP fusion protein was localized in the nucleus, indicating that OsMFS1 was a nuclear localization protein (Figures 7D–K).

### OsMFS1 Protein Physically Interacts With *OsHOP2*

**FIGURE 8:**
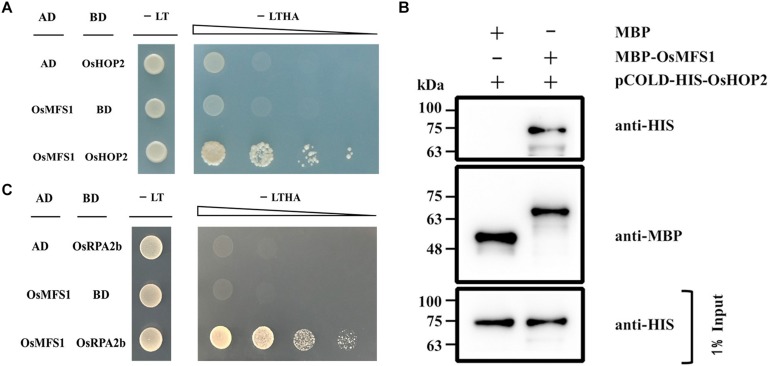
Yeast-two-hybrid (Y2H) assays and *in vitro* pull-down assay to test interactions of OsMFS1, *OsHOP2*, and OsRPA2b. **(A)** Examination of the interaction between OsMFS1 and *OsHOP2*. **(B)** *in vitro* pull-down assay was conducted to confirm the interaction between OsMFS1 and *OsHOP2*, MBP-MFS1 and pCOLD-His-HOP2 interaction *in vitro*, but not MBP itself. **(C)** Examination of the interaction between OsMFS1 and OsRPA2b. The interactions were verified by growing the yeast on the selective medium, Full-length OsMFS1 was inserted in the prey vector pGADT7 (AD). Full-length *OsHOP2* and OsRPA2b were cloned into the bait vector pGBKT7 (BD). -LT, selective medium (SD–Leu/–Trp); -LTHA, selective medium (SD–Leu/–Trp/–His/–Ade).

It was previously reported that MND1 specifically interacted with HOP2 in yeast and *Arabidopsis*, and their complex could efficiently condensate double-stranded DNA and support strand invasion (Pezza et al., 2010). OsMFS1 and MND1 are homologous gene families (Supplementary Figure S3), and to test the interactive relationship between *OsMFS1* and *OsHOP2* in rice, we performed protein interaction between OsMFS1 and *OsHOP2* by a Y2H assay. The results demonstrated that the OsMFS1 could physically interact with the *OsHOP2* (Figure 8A). To further confirm the interaction, an *in vitro* pull-down assay was conducted and proved the interaction between the OsMFS1 and *OsHOP2* (Figure 8B). In addition, we found a novel interaction between OsMFS1 and OsRPA2b by a Y2H assay (Figure 8C), the OsRPA2b was another meiotic gene, and the interaction needed further verification.

**FIGURE 9:**
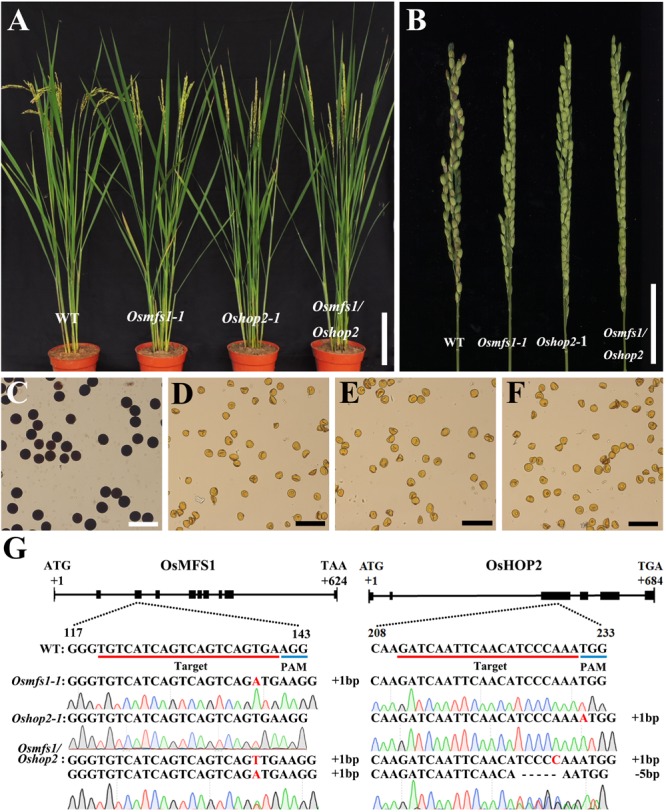
Phenotype analysis of *Osmfs1-1*, *Oshop2-1*, *Osmfs1*/*Oshop2*, and WT. **(A)** Comparison between a WT plant and an *Osmfs1-1* plant, an *Oshop2-1* plant, an *Osmfs1*/*Oshop2* plant at the heading stage. Bars = 10 cm. **(B)** Comparison of panicle between the WT and the *Osmfs1-1*, the *Oshop2-1*, the *Osmfs1*/*Oshop2* at the harvest stage. Bars = 4cm. **(C)** Viable pollen grains in a WT plant. **(D–F)** Inviable pollen grain in the *Osmfs1-1*, *Oshop2-1*, and *Osmfs1/Oshop2*. Bars = 100 μm in panels **(C–F)**. **(G)** The schematic representations of the gene structure of *OsMFS1* and *OsHOP2*, the knockout sites of *Osmfs1-1*, *Oshop2-1*, *Osmfs1/Oshop2* were verified by sequencing.

**FIGURE 10:**
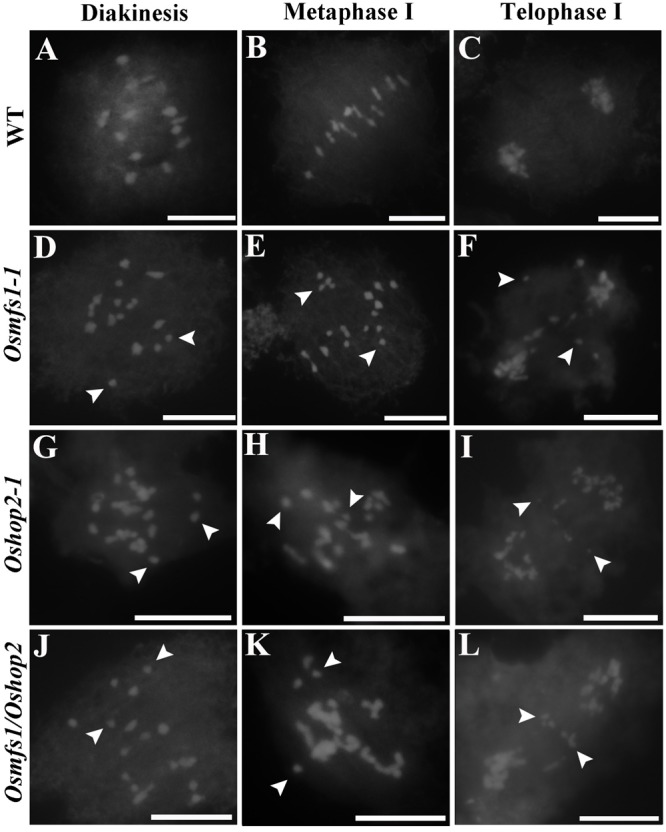
Analysis of meiotic chromosome behavior in *Osmfs1-1*, *Oshop2-1*, *Osmfs1/Oshop2*, and WT. **(A,D,G,J)** pollen mother cell in diakinesis. **(B,E,H,K)** pollen mother cell in metaphase I. **(C,F,I,L)** pollen mother cell in telophase I. The mutant *Oshop2-1* and *Osmfs1/Oshop2* shared the same chromosomal defects with *Osmfs1-1*. The white arrows point to free univalents. Bars = 50 μm.

Subsequently, to determine the link between OsMFS1 and *OsHOP2*, meiotic chromosome behavior of *Oshop2* and *Osmfs1/Oshop2* mutants were studied. We generated loss-of-function single and double mutants by CRISPR/Cas9. Fortunately, we obtained the homozygous *Oshop2-1* mutants and *Osmfs1/Oshop2* double mutants (Figures 9A,G), and phenotypic analysis results showed that the *Oshop2-1* and *Osmfs1/Oshop2* exhibited complete sterility at the harvest stage, and the pollens were inviable (Figures 9B–F). The *Oshop2-1* and *Osmfs1*/*Oshop2* shared the same chromosomal defects with *Osmfs1-1*, and the univalents were easy to distinguish (Figures 10A–L), indicating that OsMFS1 and *OsHOP2* probably act in the same pathway during meiotic DSB repair. All in all, the mutation of *OsMFS1* and *OsHOP2* led to meiotic dysplasia, and no normal pollen grains were formed. Consequently, these mutants showed complete sterility. Thus, our results confirmed that the OsMFS1 is a rice homolog of MND1 and physically interacts with *OsHOP2* to participate in DSB repair in the meiosis.

## Discussion

In this study, we showed the interaction between OsMFS1 and *OsHOP2* by Y2H and pull-down assay in rice, which were also observed in yeasts, mammals, and *Arabidopsis thaliana* (Hideo and Shirleen, 2002; Petukhova et al., 2005; Claudia et al., 2006). The *Oshop2* mutants shared the same chromosomal defects with *Osmfs1*, suggesting that the OsMFS1 and the *OsHOP2* acted in the same pathway forming heterodimeric complex to stimulate DSB repair. Therefore, *MFS1* is conserved in higher eukaryotes with common functions in different species (Supplementary Figure S2). Several studies indicated that the MND1 always interact with HOP2 to form a stable heterodimer in various species, including yeasts, *Arabidopsis thaliana*,and mammals, and both *MND1* and *HOP2* are predicted to have coiled coil domain that promotes the connection by coiled-coil interaction (Burkhard et al., 2001). The complex also physically interacts with DMC1 and RAD51 to ensure the proper synapsis of homologous chromosomes (Petukhova et al., 2005; Claudia et al., 2006), suggesting the central role of the MND1/HOP2 complex in meiotic HR. In *S. cerevisiae*, the HOP2 protein is expressed specifically during meiosis and the *hop2*-null mutant shares the same serious meiotic defects with the *mnd1* mutant: DSBs unrepaired and arrests at the meiotic pachytene stage (Leu et al., 1998; Gerton and Derisi, 2002; Zierhut et al., 2004). In mice, strand invasion activity of HOP2 protein is strong and capable of promoting D-loop formation, but no such activity is detected in the MND1/HOP2 complex, and the recombinational activity of HOP2 protein is most likely suppressed or quenched by MND1, suggesting a novel regulatory mechanism (Petukhova et al., 2005). We speculated that the recombinational activity of HOP2 in the complex might be an effective driving force to interact with and stimulate DMC1- and RAD51-mediated single-stranded DNA (ssDNA) invasion into homologous chromosomes to form a synaptonemal complex.

Previous studies showed that the MND1/HOP2 complex was the key factor to activate the invasion vitality of both DMC1 and RAD51 D-loop formation, but not the individual proteins, and the foci of DMC1 and RAD51 accumulate on single strands and the subsequent process was arrested in the *mnd1* or *hop2* mutant (Petukhova et al., 2003; Bugreev et al., 2014). Although the MND1 abrogated the recombinase activity of HOP2, it formed a new molecular interface to interact with DMC1 and RAD51. However, the function of the MND1/HOP2 complex is indispensable. In the mutant of either *mnd1* or *hop2*, the homologous chromosome pairing and DSB repairing were both abnormal and disordered (Leu et al., 1998; Gerton and Derisi, 2002; Zierhut et al., 2004). In our study, we investigated the chromosome behavior of the *Osmfs1* mutants and found that the mutants were defective in the chromosome pairings and the unpaired chromosomes were clearly observed, eventually resulting in a large number of free univalents. Due to *OsMFS1* gene mutation, the complex OsMFS1/*OsHOP2* would lose their function and the DSB would not be repaired at all. The mutants were completely sterile, and the I_2_-KI staining also showed that the mutant’s pollen was completely aborted. The MND1/HOP2 complex promoted DMC1 and RAD51 to complete the invasion of single strands whereas itswas not active itself in the formation of the D-loop (Petukhova et al., 2005). DMC1 and RAD51 have the potential ability to form D-loop. Neither of them can promote the formation of D-loop, and the MND1/HOP2 complex is needed to activate such vitality to function (Hideo and Shirleen, 2002; Petukhova et al., 2005; Bugreev et al., 2014). Although both DMC1 and RAD51 take part in forming D-loop, these two approaches are not equal. During WT meiosis, DMC1-mediated interhomolog (IH) DNA repair and the pathway appears to be predominant, whereas the pathway of RAD51-mediated intersister (IS) DNA repair has just a supportive role (Bishop et al., 1992; Cloud et al., 2012; Valerie et al., 2012). Meiotic *RAD51* is negatively regulated by *DMC1*. In *Arabidopsis*, in the presence of DMC1, the pathway DMC1-mediated would be sufficiently activated by the complex MND1/HOP2 (Marie-Therese et al., 2012; Clemens et al., 2013), whereas the pathway RAD51-mediated would only have a limited back-up function. In the absence of DMC1, the suppression of RAD51 is relieved, and the complex MND1/HOP2 is dispensable for inter-sister (IS) DNA repair (Clemens et al., 2013). Taken together, we speculated that neither DMC1-mediated nor RAD51-mediated pathways are activated during the meiosis in the *Osmfs1* mutant. This also implied that the chromosomal defects may be only part of the developmental abnormalities that caused by the mutation. How *Osmfs1* causes such defects needs further investigation. Fortunately, we also obtained *Oshop2-1* mutants and *Osmfs1*/*Oshop2* double mutants. The phenotypic identification of these mutant plants revealed that their phenotypes were consistent with *Osmfs1*, and the pollen was completely non-viable. Both the two mutants showed complete sterility. Using PI staining, it was found that the plants of both lines showed severe chromosomal defects (Figure 9); we speculate that OsMFS1 plays an important role in mediating the maturation of CO.

Recently, the interaction of *OsHOP2* and OsZIP1 was reported, indicating that *OsHOP2* play a key role in facilitating pairing of synapsis and the formation of CO (Shi et al., 2019). Obviously, not all *OsHOP2* form a complex with OsMFS1. Here we identified that the OsMFS1 protein could interact with another meiotic protein OsRPA2b through Y2H, which had not been reported before (Figure 8C). The RPA (Replication protein A) is a kind of ssDNA binding protein (SSB) and plays an essential role in multiple processes of eukaryotic DNA metabolism, including DNA replication, DNA repair, and homologous recombination in human and yeast DNA (*Saccharomyces cerevisiae*) (Johnson and O’Donnell, 2005; Machida et al., 2005). Heterotrimeric protects ssDNA and preserves the formation of hairpin (Ellen et al., 2006). The stable heterotrimer have three subunits: *RPA1* (∼70 kDa), *RPA2* (∼32 kDa), and *RPA3* (∼14 kDa). These *RPA* subunits have multiple copies in *Arabidopsis* and rice (Chang et al., 2009), *OsRPA2b* is one copy of *OsRPA2* in rice. OsMFS1 physically interact with OsRPA2b rather than other subunits, and it suggests the unknown and positive connection between OsMFS1 and OsRPA. These results indicate that these two proteins, OsMFS1 and *OsHOP2*, not only form a complex, but also possess independent functions. Our results confirmed that the phenotype of the *Osmfs1* mutant is induced by the loss of function of the OsMFS1/*OsHOP2* complex and the DSB fails to be repaired. Nevertheless, in-depth study is underway on the individual features of OsMFS1 involved in homologous recombination.

## Data Availability Statement

The raw data supporting the conclusions of this article will be made available by the authors, without undue reservation, to any qualified researcher.

## Author Contributions

JW supervised the project. JW, ZZ, CW, and JL conceived and designed the research plans. JL mapped *MFS1* and wrote the manuscript. JL, CW, and HW performed the experiments and collected and analyzed the data. HZ and WB performed the semi-thin sections. DL, YT, and YX performed real-time PCR. SY, QW, and XY generated the transgenic plants. SL, XL, and LC conducted the fieldwork. ZZ, JL, and CW supervised and complemented the writing.

## Conflict of Interest

The authors declare that the research was conducted in the absence of any commercial or financial relationships that could be construed as a potential conflict of interest.

## Footnotes

1 http://www.ncbi.nlm.nih.gov/

2 http://cbi.hzau.edu.cn/cgi-bin/CRISPR

## Supplementary Material

FIGURE S1 Phenotype analysis of WT, *Osmfs1-1*, *Osmfs1-2* (The positive plants of CRISPR/Cas9). **(A)** Comparison between a WT plant and an *Osmfs1-1* plant and an *Osmfs1-2* plant at the heading stage. Bars = 10cm. **(B–D)** Analysis of meiotic chromosome behavior in WT, *Osmfs1-1* and *Osmfs1-2* in metaphase I. The white arrows point to free univalents. Bars = 50 μm in panel **(B)**. **(E)** Seed setting rates of WT (91.80%, *n* = 174, 230, 169, respectively), *OsMFS1*/*Osmfs1* (90.57%, *n* = 170, 142, 165, respectively), *Osmfs1* (0%, *n* = 492), *Osmfs1-1* (0%, *n* = 156, 196, 160, respectively), *Osmfs1-2* (0% *n* = 177, 142, 127, respectively) at the heading stage, three panicles were counted. **(F,G)** Female fertility analysis of WT and *Osmfs1*. **(F)** The saturated pollination results showed that the seed setting rate of WT was 73.91% (*n* = 92), and the *Osmfs1* mutant was completely sterile (0%, *n* = 104), one panicle was counted. **(G)** Hoechest staining of embryo sacs showed the WT (100%, *n* = 32) develops normally while the mutant was completely sterile (0%, *n* = 27). n indicates the numbers of spikelet or embryo sac; Nd indicates No detection.

FIGURE S2 Amino acid alignment of OsMFS1 and its homologs. The sequences obtained from NCBI database and compared using Bioxm. Five species were selected including *Oryza sativa*, *Arabidopsis thaliana*, *Zea mays*, *Glycine max*, and *Nicotiana*. Amino acid similarities are shaded in pink.

FIGURE S3 Phylogenetic tree of the OsMFS1 proteins. Phylogenetic tree was constructed by MEGA 7.0 using Neighbor-Joining method, including *Sorghum bicolor*, *Zea mays*, *Panicum hallii*, *Setaria italica*, *Oryza sativa*, *Brachypodium distachyon*, *Glycine max*, *Gossypium hirsutum*, *Arabidopsis thaliana*, *Brassica napus*, *Nicotiana tabacum*, *Solanum tuberosum*, *Mus musculus*, *Homo sapiens*, *Saccharomyces cerevisiae*.

TABLE S1 Primers used in map-based cloning, RT-qPCR and plasmid construction.
