# Supplementary figures and images for: OsMFS1/OsHOP2 Complex Participates in Rice Male and Female Development
Source: Front Plant Sci. 2020 May 15;11:518. doi: 10.3389/fpls.2020.00518 (PMC7243175; doi:10.3389/fpls.2020.00518)

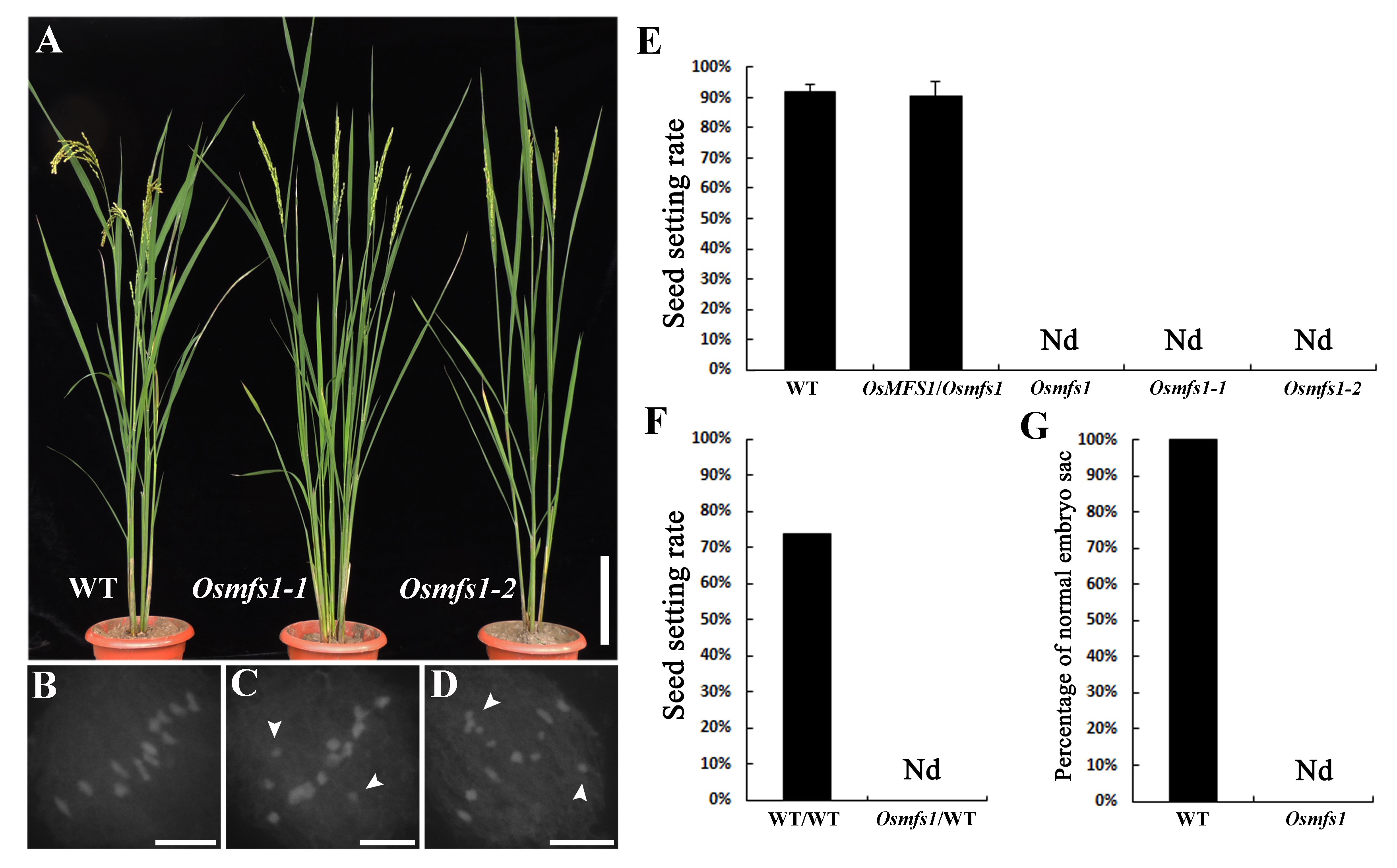

Supplement: FIGURE S1 — Phenotype analysis of WT, Osmfs1-1, Osmfs1-2 (The positive plants of CRISPR/Cas9). (A) Comparison between a WT plant and an Osmfs1-1 plant and an Osmfs1-2 plant at the heading stage. Bars = 10cm. (B–D) Analysis of meiotic chromosome behavior in WT, Osmfs1-1 and Osmfs1-2 in metaphase I. The white arrows point to free univalents. Bars = 50 μm in panel (B). (E) Seed setting rates of WT (91.80%, n = 174, 230, 169, respectively), OsMFS1/Osmfs1 (90.57%, n = 170, 142, 165, respectively), Osmfs1 (0%, n = 492), Osmfs1-1 (0%, n = 156, 196, 160, respectively), Osmfs1-2 (0% n = 177, 142, 127, respectively) at the heading stage, three panicles were counted. (F,G) Female fertility analysis of WT and Osmfs1. (F) The saturated pollination results showed that the seed setting rate of WT was 73.91% (n = 92), and the Osmfs1 mutant was completely sterile (0%, n = 104), one panicle was counted. (G) Hoechest staining of embryo sacs showed the WT (100%, n = 32) develops normally while the mutant was completely sterile (0%, n = 27). n indicates the numbers of spikelet or embryo sac; Nd indicates No detection. [file Image_1.JPEG]

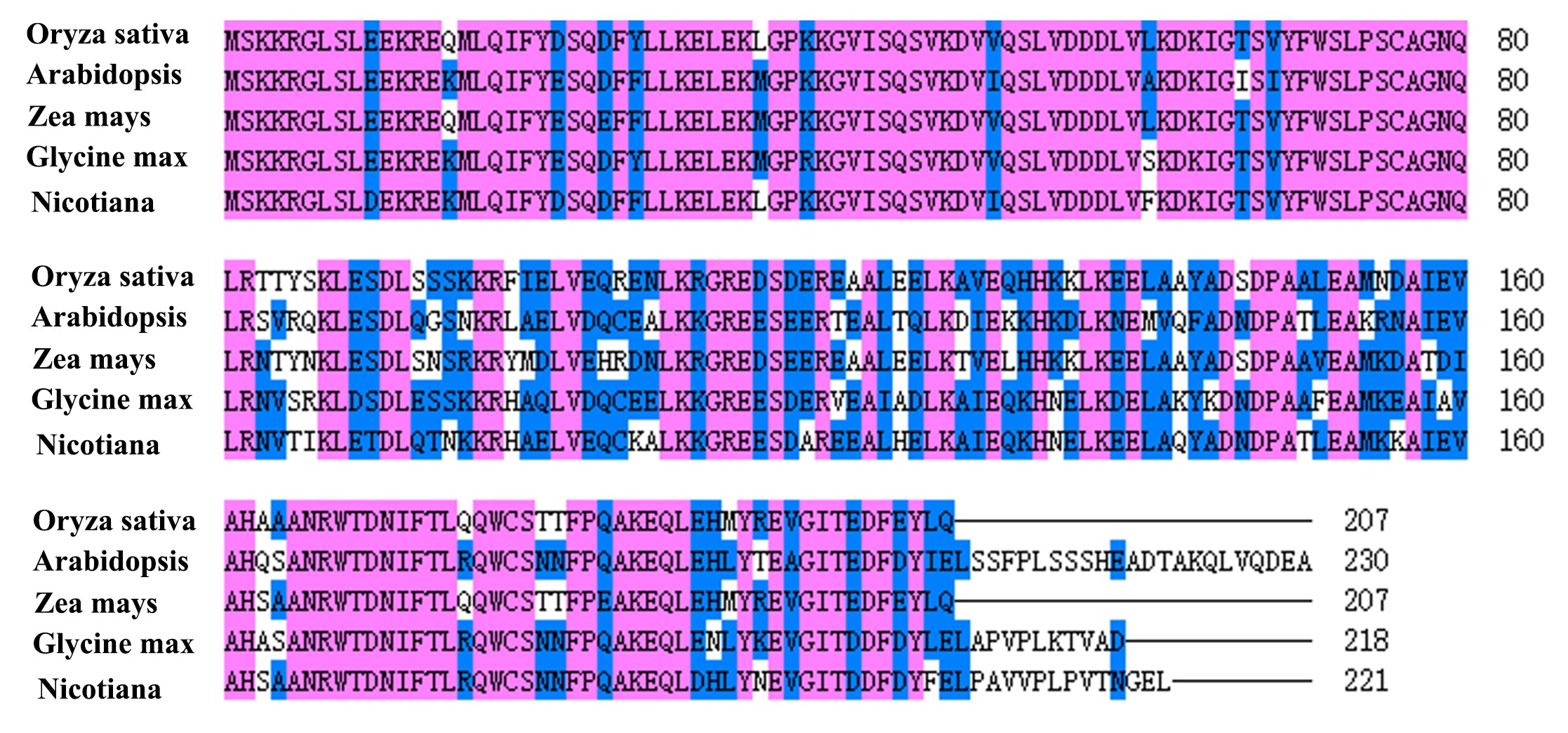

Supplement: FIGURE S2 — Amino acid alignment of OsMFS1 and its homologs. The sequences obtained from NCBI database and compared using Bioxm. Five species were selected including Oryza sativa, Arabidopsis thaliana, Zea mays, Glycine max, and Nicotiana. Amino acid similarities are shaded in pink. [file Image_2.JPEG]

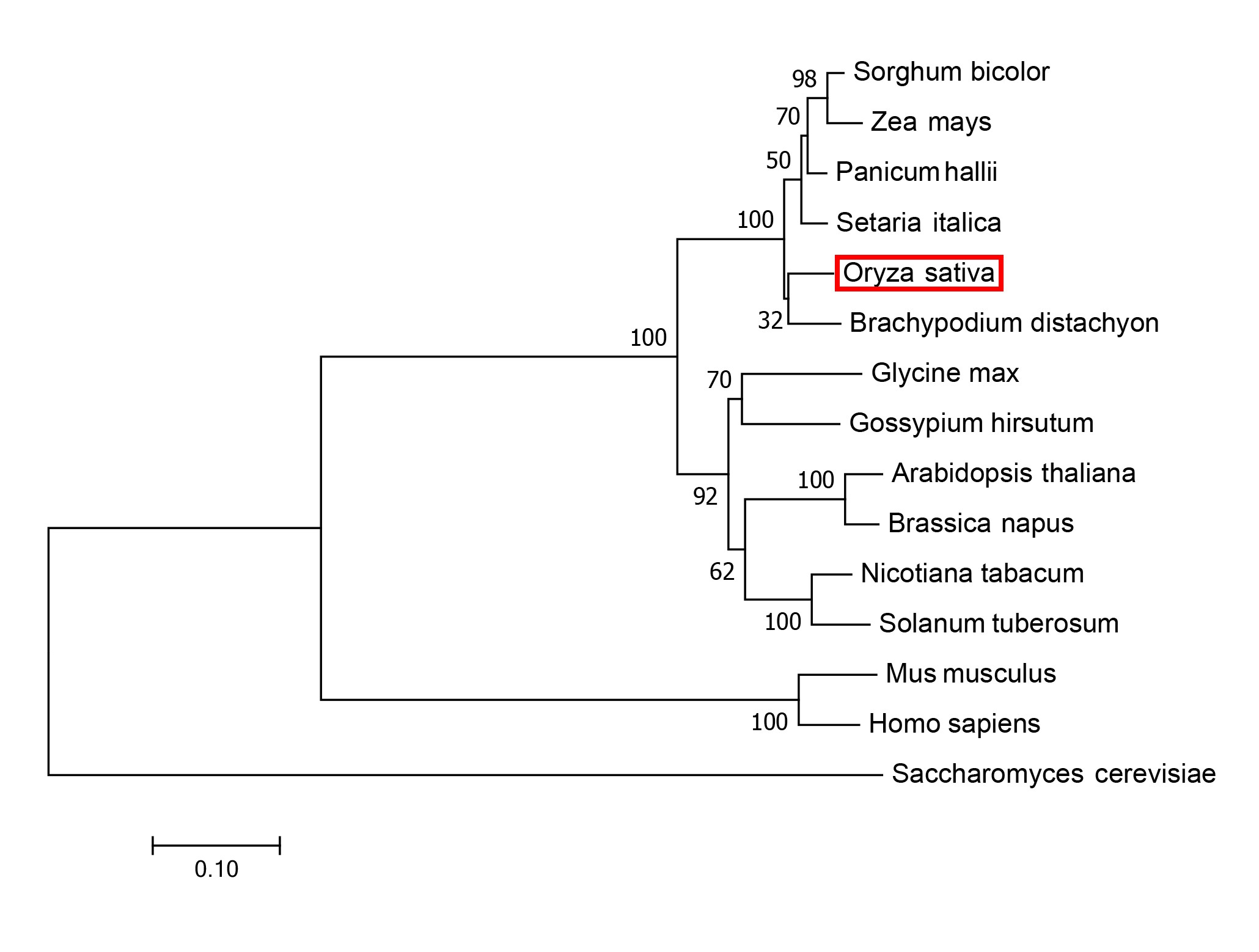

Supplement: FIGURE S3 — Phylogenetic tree of the OsMFS1 proteins. Phylogenetic tree was constructed by MEGA 7.0 using Neighbor-Joining method, including Sorghum bicolor, Zea mays, Panicum hallii, Setaria italica, Oryza sativa, Brachypodium distachyon, Glycine max, Gossypium hirsutum, Arabidopsis thaliana, Brassica napus, Nicotiana tabacum, Solanum tuberosum, Mus musculus, Homo sapiens, Saccharomyces cerevisiae. [file Image_3.JPEG]
