# Supplementary material for: OsMFS1/OsHOP2 Complex Participates in Rice Male and Female Development
Source: Front Plant Sci. 2020 May 15;11:518. doi: 10.3389/fpls.2020.00518 (PMC7243175; doi:10.3389/fpls.2020.00518)
Supplement: TABLE S1 — Primers used in map-based cloning, RT-qPCR and plasmid construction. [file Table_1.pdf]

Supplemental Table S1 Primers used in map-based cloning, RT-PCR and plasmid construction

引物名称	引物序列		备注
RM23662	F	GAGAGGACGATGGCACTATTGG	Map-based cloning
	R	CGAGGAACTTGATTCGCATGG	
RM23916	F	GATGGGTTGGGTGGGTTAGG	
	R	GTCCAGTGATAAGCCATGCTTGC	
NN9S-L4	F	GAATTGCTTAGGTTCCCTTGATT	
	R	ACAGGGACACCACAAAGATTATA	
NN9S-L7	F	CATTGTTGTGGATTATGACCCTT	
	R	CTGGAGCTGCTTTGTAAGAAAG	
NN9S-R7	F	AGTTGGAATGCGATCATACTATCT	
	R	GGCATGTGCAAACATACAATTTG	
NN9S-R2	F	TTACCAAGTACTTGTGACAAACG	
	R	GCTCGTGGACTTAAAAACAAGAA	
JY-10	F	GGATGACATAGAGGTAGAAGTG	
	R	TGATGCTGTTACACACA	
OsMFS1-DL	F	TGCTGGAAATCAGCTGAGGA	Real-Time PCR
	R	TCAGAGTCCTCTCTGCCTCT	
OsHOP2-DL	F	CCTCAGCTTCGTCAATGAGC	
	R	TGCGGTCTTCTTGAGGCTAA	
CRISPR-MFS1	F	TGTCATCAGTCAGTCAGTGA	CRISPR/Cas9
	R	TCACTGACTGACTGATGACA	
CRISPR-HOP2	F	GATCAATTCAACATCCCAAA	
	R	TTTGGGATGTTGAATTGATC	
AD-MFS1	F	GGAGGCCAGTGAATTCATGTCGAAGAAGAGGGGTCTT	Y2H-AD
	R	TCATCTGCAGCTCGAGTTACTGCAGATATTCGAAATC	
BD-MFS1	F	CATGGAGGCCGAATTCATGTCGAAGAAGAGGGGTCTT	Y2H-BD
	R	TAGTTATGCGGCCGCTGCAGTTACTGCAGATATTCGAAATC	
AD-HOP2	F	GGAGGCCAGTGAATTCATGCCTCCCAAATCTGATAGC	Y2H-AD
	R	TCATCTGCAGCTCGAGTCAGCGGGATACTTTCCTTCT	
BD-HOP2	F	CATGGAGGCCGAATTCATGCCTCCCAAATCTGATAGC	Y2H-BD
	R	TAGTTATGCGGCCGCTGCAGTCAGCGGGATACTTTCCTTCT	
GUS-MFS1	F	CCATGATTACGAATTCGGTGATGGTGCAAAGTAGGC	GUS Staining
	R	CTCAGATCTACCATGGTCTGCCCTAGGATTGCCGTA	
PN580-MFS1	F	CGGAGCTAGCTCTAGAATGTCGAAGAAGAGGGGTCTT	Subcellular localization
	R	TGCTCACCATGGATCCCTGCAGATATTCGAAATC	

Supplemental Table S1 | Primers used in map-based cloning, RT-qPCR and plasmid construction.
